# The EAT-Lancet diet associated cardiovascular health parameters: evidence from a Brazilian study

**DOI:** 10.1186/s12937-024-01021-4

**Published:** 2024-10-01

**Authors:** Rosa Sá de Oliveira Neta, Severina Carla Vieira Cunha Lima, Maria Fernanda Araújo de Medeiros, David Bruno Melo Araújo, Nicole Bernardi, Armando Augusto Noberto Galdino de Araújo, Michelle Cristine Medeiros Jacob, Adélia da Costa Pereira de Arruda Neta, Dirce Maria Lobo Marchioni, Clélia de Oliveira Lyra, Angelo Giuseppe Roncalli da Costa Oliveira

**Affiliations:** 1 Postgraduate Program in Collective Health, Federal University of Rio Grande do Norte, Natal, Rio Grande do Norte Brazil; 2 Department of Nutrition, Federal University of Rio Grande do Norte, Natal, Rio Grande do Norte Brazil; 3 Postgraduate Program in Social Sciences, Federal University of Rio Grande do Norte, Natal, Rio Grande do Norte Brazil; 4 Center for Studies and Research in Food, State University of Campinas, Campinas, São Paulo, Brazil; 5 Department of Nutrition, School of Public Health, University of São Paulo, São Paulo, Brazil

**Keywords:** Cardiovascular diseases, Cardiometabolic parameters, EAT-Lancet diet, Sustainable diets, Ultraprocessed foods

## Abstract

**Background:**

The EAT-Lancet diet is a diet aimed at promoting population and planetary health from the perspective of sustainable diets in terms of environmental and health aspects. This study aimed to assess the association between adherence to the EAT-Lancet diet and cardiometabolic risk factors among adults and elderly individuals in a capital city in the northeastern region of Brazil.

**Methods:**

This is an analytical cross-sectional observational study from a population-based sample conducted between 2019 and 2020, involving 398 non-institutionalized adults and elderly people, of both sexes from “Brazilian Usual Consumption Assessment” study (Brazuca-Natal). There was a 38% response rate due to the suspension of data collection due to the covid-19 pandemic, but According to the comparative analysis of socioeconomic and demographic variables between the surveyed and non-surveyed sectors, losses were found to be random (*p* = 0.135, Little’s MCAR test). Socioeconomic and lifestyle data, anthropometric measurements, and dietary consumption were collected. We used the Planetary Health Diet Index (PHDI) and the Cardiovascular Health Diet Index (CHDI) for cardiovascular health to assess adherence to the diet’s sustainability. The evaluated cardiometabolic parameters included fasting blood glucose, triglycerides, total cholesterol, HDL-C, LDL-C, and systolic and diastolic blood pressure measurements. We also assessed the presence of type 2 diabetes mellitus, arterial hypertension, and dyslipidemia. For the data analyses, sample weights and the effect of the study design were taken into account. Pearson’s chi-square test was used to evaluate the statistical significance of frequencies. Multiple linear regression models assessed the associations between PHDI and CHDI and its components and the cardiometabolic parameters.

**Results:**

The mean PHDI was 29.4 (95% CI 28.04:30.81), on a total score ranging from 0 to 150 points and the mean CHDI was 32.63 (95% CI 31.50:33.78), on a total score ranging from 0 to 110 points. PHDI showed a significant positive association with the final CHDI score and components of fruits, vegetables, and legumes, and a negative association with Ultra-processed Food (UPF) (*p* < 0.05). Notably, among the most consumed UPF, the following stand out: “packaged snacks, shoestring potatoes, and crackers” (16.94%), followed by margarine (14.14%). The PHDI exhibited a significant association with diabetes and dyslipidemia, as well as with systolic blood pressure, total cholesterol, and LDL-C.

**Conclusions:**

The results suggest that adopting the EAT-Lancet diet is associated with the improvement of key cardiovascular health indicators.

## Introduction

Non-communicable chronic diseases (NCDs) account for 70% of deaths, 45% of which are caused by cardiovascular diseases (CVD), equivalent to more than 17 million deaths worldwide [1, 2]. Approximately 75% of CVDs are preventable, requiring adequate management of cardiometabolic risk factors (hypertension, obesity, diabetes, and dyslipidemia) and behavioral factors associated with smoking, alcohol consumption, sedentary lifestyle, and unhealthy diet, characterized by high intake of fats, sodium, refined carbohydrates, and sweetened beverages. In addition to health implications, examining how these diets contribute to environmental degradation is important, given that dietary choices are essential determinants of both human health and environmental sustainability [3, 4].

There is a well-established intrinsic relationship in analyzing the consumption of sustainable diets and their impact on health, considering that dietary patterns affect the environment and vice versa [5, 6]. Nutritional recommendations for preventing CVDs advocate limiting the intake of added sugars, saturated fats, sodium, and red meats, encouraging their replacement with high-quality vegetable protein sources as a protective factor [7–10]. On the other hand, sustainable diets consist of vegetables, fruits, whole grains, legumes, moderate to low amounts of seafood and poultry, and little to no red meat, refined grains, added sugars, and starchy vegetables [11]. According to the Food and Agriculture Organization (FAO) and World Health Organization (WHO) they are diets with low environmental impact that contribute to food and nutritional security, and promote a healthy life for future generations, must protect and respect biodiversity and ecosystems, be acceptable and accessible, from a cultural and economical, be nutritionally adequate, safe and healthy, and optimize natural and human resources [12].

It is important to state that, even with a significant overlap between health-promoting and environmentally sustainable diets, the main discourse of planetary health theory, aligning a healthy diet with a sustainable planet; however, is not always achievable. A nutritionally adequate diet may have a high environmental impact, whereas a diet with lower ecological impacts may be nutrient-poor [6].

The benefits of consuming a sustainable diet for cardiovascular health occur mainly through the replacement of saturated fats with unsaturated fats and increased dietary fiber intake, both mechanisms leading to a reduction in the absorption of LDL-c and glucose [13, 14], recognized risk factors for CVD [15, 16]. In addition to improving lipid profiles, regular fiber consumption may promote weight loss and reduce visceral adiposity due to the satiety effect [17, 18].

It is essential to assess sustainable diets in a context of inequalities among countries, as their accessibility is a concerning issue, considering that individuals with high adherence to this diet exhibit higher food costs [19, 20], and access is only possible through the adoption of different combinations of consumed foods [11]. It is urgent to adhere to a diet that is simultaneously healthy, accessible, and ecological to face the growing environmental crises [21], food insecurity, and the global syndemic [22].

In early 2019, the EAT-Lancet Commission released a scientific report titled “Healthy Diets from Sustainable Food Systems,” outlining sustainable dietary practices. Within this report, they introduced the “planetary health diet” (PHD), a suggested dietary framework aimed at promoting both human and planetary well-being. In summary, this diet this diet emphasizes eating mostly vegetables, fruits, whole grains, legumes, nuts, and unsaturated oils. It encourages a moderate intake of seafood and poultry while minimizing or avoiding red meat, processed meat, added sugar, refined grains, and starchy vegetables [11]. These dietary recommendations are formulated considering the impacts on human health and the environment stemming from our food systems but does not address all the dimensions included in actually sustainable diets (i.e., economic and sociocultural aspects) [11, 20].

Synergism between risk (or protective) factors for cardiovascular health and planetary health can only be identified in the study of dietary patterns, as they provide a more comprehensive analysis that is consistent with current recommendations in the scientific literature. Dietary patterns are classified into two approaches: the first, called a *priori*, is based on prior knowledge of the favorable and unfavorable effects of dietary constituents (e.g. using the Diet Quality Index). The other approach, a *posteriori*, is based on the dietary data obtained. The main techniques in this latter approach are principal component analysis, followed by factor analysis, and this approach requires statistical modeling [23].

In light of the challenge of measuring adherence to healthy and sustainable diets, it is worth stressing that in 2021, the Planetary Health Diet Index (PHDI) was developed, a *priori* Brazilian index that assesses diet quality and considers the characteristics proposed by the EAT-Lancet reference diet. The development, application, and validation have been previously described [24, 25]. Notably, this index has demonstrated a significant association with the improvement of cardiometabolic factors and the prevention of CVD [26] and the PHDI was associated with lower risk of deaths from cardiovascular diseases [27].

The Cardiovascular Health Diet Index (CHDI) score remained associated with a higher overall dietary quality and preventing cardiovascular disease, such as being positively associated with carbohydrates, total protein, vegetable protein source, PUFA and fiber intake, while it has been negatively associated with total fat, MUFA, saturated fat, cholesterol, added sugars, and sodium [28]. The CHDI stands out in studies examining the association between sustainable diets and cardiometabolic risk [3, 27, 29, 30] by assessing the consumption of ultra-processed foods (UPF) using the NOVA classification [31UPF are critically defined based on the extent and purpose of processing, typically presenting high energy density, being rich in sugar and fat, and low in fiber, vitamins, and minerals, and are associated with the development of CVD [28, 32, 33]. Furthermore, these foods can potentially be detrimental to achieving the goals of sustainable food systems in terms of minimizing environmental impacts and prioritizing the production of nutritious and biodiverse foods [34].

Expanding research using the CHDI methodology can provide valuable insights into the cardioprotective benefits of sustainable diets, serving as a crucial tool for globally analyzing dietary habits and developing strategies to reduce UPF consumption. Therefore, the objective of the present study was to assess the association between adherence to the EAT-Lancet diet and CHDI with cardiometabolic risk factors in adults and elderly individuals in a capital city in the northeastern region of Brazil.

## Materials and methods

### Study design

This is an analytical cross-sectional observational study conducted between 2019 and 2020, involving 398 non-institutionalized adults and elderly people, of both sexes. The analysis of data from the first phase of the study “Food Insecurity, Health and Nutrition Conditions in Adult and Elderly Population of a Northeastern Brazilian Capital: Brazuca Study (Brazilian Usual Consumption Assessment) conducted in Natal, Rio Grande do Norte (Brazuca Natal Study). The city of Natal was chosen to assess the association between sustainability and cardiovascular diseases using an index that evaluates UPF intake, as it leads in the availability of ultra-processed foods according to a national dietary survey—NDS-HBS (in Portuguese Inquérito Nacional de Alimentação—Pesquisa de Orçamentos Familiares—INA/POF) – 2017–2018, considering the states in the North and Northeast regions [35].

The sampling plan considered a probability sampling by conglomerates in two stages (census tracts and households). The census tracts were randomly selected with probability proportional to their size (number of households). The selection aimed to obtain a minimum of 258 interviews for each of the four strata of sex and age: adults (20 to 59) and elderly individuals (60 years or older) of both sexes. The minimum size of 258 individuals in each stratum allowed estimating a prevalence of 50% with an error of 8% and a confidence level of 95%. The outlining effect (*deff)* was 1.5, and 15% was added as a non-response and closed households rate. The estimated total sample size was 1,032 people (258 individuals * 4 strata).

Due to the COVID-19 pandemic, data collection, began in June 2019 and was suspended in March 2020, resulting in data collection from 411 individuals. For the current study, 398 individuals with information on dietary consumption were included, of whom 111 had results of biochemical parameters, with a sample power of 80% calculated using the OpenEpi program, considering a finite population, a frequency of 25% for cardiovascular risk factors, an estimation error of 10%, and a design effect of 2.5%. There was a 38% response rate due to the suspension of data collection due to the covid-19 pandemic, but According to the comparative analysis of socioeconomic and demographic variables between the surveyed and non-surveyed sectors, losses were found to be random (*p* = 0.135, Little’s MCAR test). This indicating that the sample collected is equivalent to that initially planned and does not compromise the population representativeness of the study.

The measures that were taken into account sample weights and the effect of the study design were taken by the researchers to mitigate the effects and biases in the research as for the data analyses.The weighted sample size (n^pond^) was calculated by calibrating the sample weights so that the estimates produced coincide simultaneously with the total number of households and the total population by sex and age, obtained from exogenous sources, in the case of the Brazuca-Natal Study, from the Brazilian Institute of Geography and Statistics – BIGS (in Portuguese Instituto Brasileiro de Geografia e Estatística - IBGE) (www.ibge.gov.br). To compose the database for complex samples, the Primary Sampling Unit (PSU) was defined as the census tract number.

To determine the sample weights, the probabilities of each individual associated with the first and second stages of the draw (census tract and household, respectively) were first calculated. The first probability was given by dividing the number of households in the sector, obtained from the BIGS, by the sampling interval (total number of households in the municipality divided by the total number of sectors drawn). The second probability, relating to the second stage of the draw, was given by dividing the estimated number of individuals per household, considering each stratum of the sample (adults and the elderly), by the total number of households in the sector. Thus, each individual had two associated probabilities: (1) the probability of the first stage (census tract) and (2) the probability of the second stage (household). To obtain the sample weight of each individual, taking into account the survey’s complex sampling procedure, the inverse of the product of these two probabilities was calculated.

The variables considered to have the greatest weight in the study were those related to dietary consumption and biochemical parameters (fasting glucose, total cholesterol, HDL-c, LDL-c, and triglycerides). Data from the study showed that using a deff of 1.5 to estimate the proportion of participants who provided dietary consumption data by sex and age group resulted in proportions ranging from 16.48 (elderly males) to 181.26 (adult females). Intervals for estimates by sex and age regarding the sample weights of participants who had biochemical parameters ranging from 60.47 (elderly males) to 665.16 (adult females).

In the Brazuca Natal Study, adjustments were made to verify the potential of the sample according to the initial planning, given the singularities presented due to the Covid-19 pandemic, in order to maintain the reliability of the data and the representativeness of the sample for the city of Natal-RN.

The research adhered to the norms and regulatory standards outlined in the Resolution of the National Health Council for studies involving human subjects No. 466/2012 and approved by the Research Ethics Committee of the Onofre Lopes University Hospital/Federal University of Rio Grande do Norte (CAAE No. 96294718.4.2001.5292, Approval No. 3,531,721). Each participant volunteered and provided their signature on an informed consent form that detailed the study’s risks and benefits.

### Sociodemographic Variables

The sociodemographic data were collected using the Epicollect version 5^®^ software: sex (male and female), age group (adult and elderly), education level (illiterate, completed primary education, completed secondary education, and completed higher education) and per capita household income (≤ 1 minimum wage or > 1 minimum wage).

### Behavioral variables

The level of physical activity, alcohol consumption and smoking were considered as behavioral variables. In terms of physical activity levels, individuals were categorized as “sedentary” if they did not participate in any physical activity, or as “physically active” if they undertook ≥ 150 min/week of moderate activity or ≥ 75 min/week of vigorous activity, as per the criteria outlined in the International Physical Activity Questionnaire - IPAQ [36]. Regarding alcohol consumption, weekly ethanol consumption of ≥ 140 g for women and ≥ 210 g for men was considered, and it was dichotomized as “yes” or “no” [37]. A “smoker” was considered to be someone who used tobacco regularly, and a “non-smoker” was someone who had already smoked (at least 100 cigarettes) and had stopped smoking or had never used tobacco-derived substances [38].

### Dietary assessment

The evaluation of dietary intake involved the use of a 24-hour dietary recall (R24h), which was used to calculate the PHDI and CHDI [24]. The R24h detailed all foods consumed by the participant on the previous day and was conducted using a structured script provided by the Globodiet^®^ software (39], which guides a five stages interview: participant’s general information; a quick list of foods and recipes; details of food/recipes and quantities consumed, primarily using a photographic manual; control of food and nutrient quantities; and information about the use of dietary supplements [40].

The food measurements were then converted into nutrients using the Brazilian Food Composition Table - University of São Paulo (TBCA-USP), version 6.0 [41], integrated into the Globodiet^®^ software.

### Calculation of the planetary health diet index (PHDI)

The PHDI is an a priori index that assesses diet quality based on the recommendations of the EAT-Lancet Commission Report. This index has been described, applied, and validated in prior studies [24, 26–28]. In brief, it is a multidimensional index that features a gradual scoring system and interchangeable groups based on the intake of the 16 components it encompasses. The higher the score, the greater the adherence to the sustainable diet. The PHDI considers all food groups and the ranges and midpoint scores proposed by the EAT-Lancet (expressed in grams per day g/day and kcal/day), calculated as their energy contribution to a of 2500 kcal/day reference diet.

The individual scoring of the PHDI is based on what was described by Cacau et al. (2021) [24]. Firstly, mixed recipes were identified and decomposed into their ingredients according to standardized homemade recipes available in the national literature [42], and all foods were allocated to their respective components. The score for each component is calculated based on a caloric intake rate between the sum of all foods classified in the component regarding caloric value and the sum of the calories of all foods included in the index. Adherence to the PHDI was previously described for the population of Brazuca-Natal [43].

The PHDI comprises sixteen components, divided into the following four categories: adequacy, optimum, ratio, and moderation. A maximum of 5 or 10 points can be assigned, resulting in a total proportional score ranging from 0 to 150 points. All the components are scored between 0 points and 10 points, except for the “Ratio” component, which has a maximum score of 5 points and has a certain minimum intake level which, if exceeded, obtains a lower score; the same occurs with the “Optimum” component. A higher score is associated with a reduced consumption of foods categorized under “Moderation”, while the inverse holds true for the foods falling under “Adequacy”. This underscores the importance of promoting the consumption of specific foods to foster a healthy and sustainable diet. Alcoholic beverages were not included, as they are generally not counted in planetary health diet scores [24].

### Calculation of cardiovascular health diet index (CHDI)

**Fig. 1:**
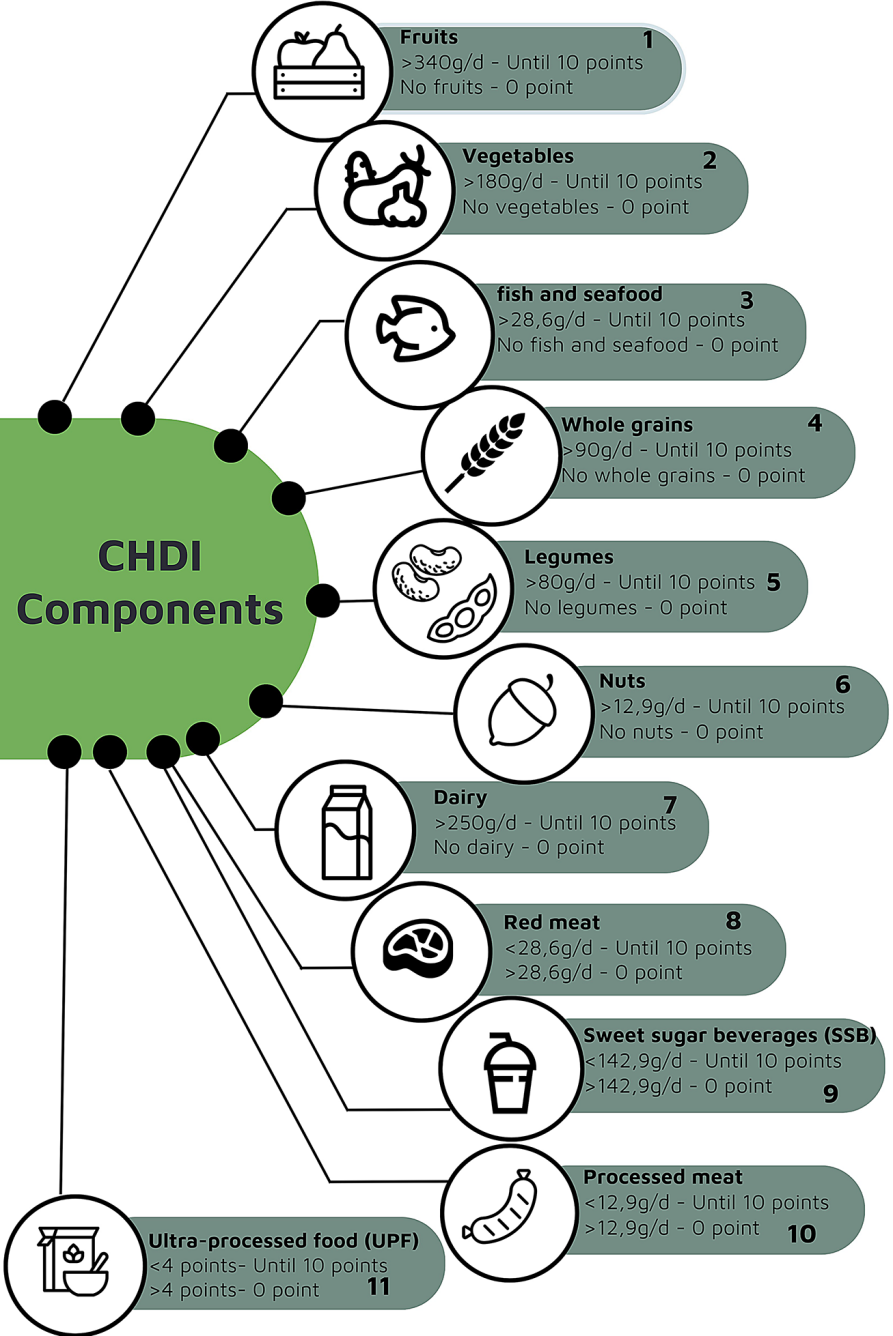
Metrics used by the CHDI. Source: Adapted from Cacau et al., 2022 [28]

The CHDI is an index based on the American Heart Association (AHA) recommendations and adapted to the Brazilian context. It is structured on eleven dietary intake metrics to assess cardiovascular health [28]. Each item the CHDI meets is scored proportionally to the consumption, which can reach up to 10 points, meaning it ranges from 0 to 110 points. The higher this score, the better the individual cardiovascular health. The parameters for each metric can be seen in Fig. 1.

According to Fig. 1, what sets the CHDI apart from the dietary recommendations of the AHA [30] is that the CHDI follows the current recommendations of the NOVA food classification, which are focused on food groups rather than specific nutrients, as the AHA did when assessing sodium and saturated fat intake [28].

To classify the UPF, the CHDI considered the NOVA Score [44], which is a tool that includes three major groups of UPF divided into 23 subgroups, ranging from 0 to 23 points. Each food included in a food subgroup is equivalent to 1 point. The first group refers to sugar-sweetened beverages (traditional or diet soda; boxed or canned fruit juice; powdered drink mix chocolate milk like tea-based beverage; and fruit or chocolate-flavored yogurt). The second group includes products that replace or accompany meals (sausage, hamburger, or nuggets; ham, salami, or mortadella; industrialized bread; margarine; frozen french fries from fast-food big chains; frozen pizza from big chains and frozen lasagna or other ready-made frozen dishes). Lastly, there is the group of snacks (packaged snacks, shoestring potatoes, or crackers; sweet cookies with or without filling; packaged cakes; cereal bars; branded ice cream or popsicles; chocolate bars or candies; and sweetened breakfast cereal Each subgroup consumed corresponds to 1 point in this score [44].

These UPFs were identified as those with the highest participation in the estimated daily energy intake based on NDS-HBS – 2008–2009 [45]. Each item consumed by the participant in the 24 h was initially categorized as ultraprocessed or non-ultraprocessed, according to the NOVA classification, to determine the contribution of UPF to the previous day’s food consumption [46].

### Cardiometabolic risk factors

The diagnosis of dyslipidemia was conducted considering the classification of the lipid profile as defined in the latest update of the Brazilian Guideline for Dyslipidemia [47]. Participants self-reported the presence of type 2 diabetes mellitus (T2DM) and arterial hypertension.

The clinical and biochemical markers measured were: Diastolic blood pressure (DBP) and systolic blood pressure (SBP) in mmHg, fasting blood glucose (cutoff point for change if ≥ 125 mg/dL), total cholesterol (cutoff point for change if ≥ 200 mg/dL), high-density lipoprotein cholesterol (HDL-c) (cutoff point for change if < 40 mg/dL), low-density lipoprotein cholesterol (LDL-c) (cutoff point for change if ≥ 130 mg/dL), and triglycerides (cutoff point for change if ≥ 130 mg/dL).

The participant’s blood pressure was measured while seated, with both feet on the floor and their back supported by the chair. The measured arm was supported in a way that allowed it to be aligned with the heart. Measurements were taken on the left arm in duplicate, with a 5-minute interval between them. The average of the two measurements was used to obtain SBP and DBP. An OMRON automatic arm blood pressure monitor (HEM 7122) was used for measurement.

For the analysis of biochemical tests, the participants underwent venipuncture in the peripheral vein in the morning, performed by a trained nursing technician, after fasting for up to 12 h. A 10 mL blood sample was collected and divided into tubes with and without anticoagulants, following specific procedures tailored to each analysis. Out of these, 4 mL were allocated for the analysis of total cholesterol, fractions, and fasting blood glucose, conducted by a reference laboratory in the municipality of Natal contracted for this purpose.

Enzymatic methods were used to analyze of fasting blood glucose and total cholesterol (colorimetry). HDL-c levels were measured using a homogeneous enzymatic colorimetric assay. LDL-c values were derived utilizing the Friedewald formula [LDL-c = Total cholesterol – HDL-c + (Triglycerides/5)], as recommended by the Brazilian Society of Cardiology [48]. All these analyses were performed using automated methods (COBAS 6000 - Roche^®^ Professional Diagnostics, Risch-Rotkreuz, Switzerland).

### Anthropometric assessment

The Body Mass Index (BMI) was computed by dividing weight (in kilograms) by the square of height. (m²) [49]. For weight measurement, a portable electronic scale (Líder^®^ model P200M) was used, with a capacity of 200 kg and an accuracy of 50 g. Height was measured with a portable stadiometer (Avanutri^®^) ranging from 20 cm to 210 cm with 0.1 cm graduation, fixed on a base with a stabilizer to lean against the wall for more excellent stability of the measuring rod. Measurement was taken with the participant standing barefoot, touching the head, hips, and heels against the device’s base, with eyes fixed on the horizontal plane. We considered cut-off points established by the World Health Organization (WHO) for adults (1995) [50] and by Lipschitz (1994) [51] for older adults.

### Data analysis

We employed the Statistical Package for the Social Sciences (IBM SPSS Statistics 22) software for statistical analysis. Methodological adjustments were made to ensure that the information generated from the subsample could provide the initially planned information. Therefore, we employed the complex sampling model, incorporating the sample design and weighting to calculate population estimates for adults and older adults in Natal-RN, Brazil. A weighted sample size (n^pond^) was used for these analyses.

In the descriptive analyses of sample characterization, frequencies were calculated for categorical variables according to the PHDI terciles. Pearson’s chi-square test assessed the statistical significance of differences between frequencies, respectively. Means and standard deviations of total scores and components of PHDI and CHDI were calculated with a 95% confidence interval (95% CI).

The frequency (%) of consumption of foods included in the NOVA score for UPF consumption was calculated. We investigated the variation in the average percentage of calories from ultra-processed foods relative to the score variation and presented continuously to assess the relationship between the NOVA score for UPF consumption and the proportion of these foods in the diet. Finally, total calories consumed, calories from UPF, and the percentage of total calories from these foods were calculated.

We developed multiple linear regression models to pinpoint factors that could serve as predictors (CHDI, clinical, and biochemical parameters) of adherence to PHDI in its continuous form (dependent variable) and to estimate the association between cardiovascular risk factors and the diet. The models were evaluated for residual symmetry, homoscedasticity of variances, and outliers. The models were adjusted for potential confounding factors were performed to assess the relationship between adherence to PHDI and outcomes, as age, BMI, and per capita income. These adjustment variables were determined from bivariate analysis, initially identifying those sociodemographic and lifestyle variables associated with PHDI (*p* < 0.2) and subsequently selecting those variables that could have an impact based on current literature. Data were considered significant when *p* ≤ 0.05.

## Results

Data from 398 individuals of both sexes were collected. Table 1 presents the characteristics of the confirmed population and their scores related to PHDI adherence terms.

**Table 1:** Characteristics of individuals included in the study and their scores related to the terciles of adherence to the Planetary Health Diet Index. Brazuca-Natal Study, Brazil (2019–2020)

Variables	Planetary health diet index (PHDI)			*p*-value*
	1st tercile (≤ 24.36 points )	2nd tercile (24.37–33.62 points)	3rd tercile (> 33.63 points)	
Age group, *n*; *n*^pond^ (%)**				
Elderly	55; 1433.76 (29.9)	72; 1450.24 (30.2)	59; 1911.68 (39.9)	0.064
Adults	77; 13034.14 (36.6)	61; 10265.82 (28.8)	74; 12358.52 (34.7)	
**Sex**,** n; n**^**pond**^(%)**				
Female	81; 9936.46 (37.6)	80; 7975.60 (30.2)	70; 8535.80 (32.3)	0.212
Male	51; 4531.44 (32.4)	53; 4201.90 (30.0)	63; 5272.96 (37.6)	
**Education level**,** n; n**^**pond**^(%)**				
Illiterate	9; 741.52 (47.9)	8; 379.02 (24.5)	10; 428.46 (27.7)	0.735
Completed Primary Education	65; 6212.34 (41.1)	60; 4185.56 (27.7)	49; 4712.80 (31.2)	
Completed Secondary Education	36; 4828.10 (32.6)	40; 4729.24 (31.9)	43; 5273.00 (35.6)	
Completed Higher Education	22; 2685.94 (30.0)	25; 2883.68 (32.2)	31; 3394.50 (37.9)	
**Per capita household income**,** n; n**^**pond**^(%)**				
≤ 1 Minimum wage	81; 8931.22 (43.9)	57; 5767.42 (28.3)	51; 5652.04 (27.8)	**0.027**
> 1 Minimum wage	48; 5174.16 (27.5)	74; 6047.56 (32.2)	77; 7563.50 (40.3)	
**Body Mass Index (BMI - kg/m²)**,** n; n**^**pond**^(%)**				
Overweight	41; 3476.92 (36.2)	45; 3493.42 (36.4)	36; 2636.54 (27.4)	**0.036**
Eutrophy	90; 10842.68 (35.3)	88; 8684.08 (28.3)	97; 11172.22 (36.4)	
**Physical activity level**,** n; n**^**pond**^(%)**				
Sedentary	99; 10018.80 (33.6)	108; 9409.16 (31.6)	104; 10348.32 (34.8)	0.335
Physically active	33; 4449.10 (41.7)	25; 2768.34 (25.9)	29; 3640.44 (32.4)	
**Alcohol consumption**,** n; n**^**pond**^(%)**				
Yes	62; 7085.60 (39.6)	47; 5734.360 (32.0)	50; 5075.26 (28.4)	0.210
No	69; 7201.04 (32.2)	86; 6443.14 (28.8)	83; 8733.50 (39.0)	
**Smoking**,** n; n**^**pond**^(%)**				
Smoker	14; 1845.54 (49.6)	10; 790.96 (21.2)	7; 1087.56 (29.2)	0.296
Non-Smoker	117;12441.10 (34.0)	123; 11386.54 (31.2)	126;12721.20 (34.8)	
**Arterial hypertension**,** n; n**^**pond**^(%)**				
Yes	54; 4300.88 (39.1)	50; 2817.88 (25.6)	54; 3872.44 (35.2)	0.128
No	76; 9804.50 (34.0)	80; 9145.40 (31.7)	78; 9903.36 (34.3)	
**Diabetes mellitus**,** n; n**^**pond**^(%)**				
Yes	21; 1730.22 (36.0)	25; 1384.22 (28.8)	28; 1697.30 (35.3)	0.188
No	108;12506.98 (35.7)	106;10463.72 (29.8)	104; 12078.50 (34.5)	
**Dyslipidemia**,** n; n**^**pond**^(%)***				
Yes	21; 2026.84 (29.1)	26; 2274.020 (32.6)	32; 790.96 (30.8)	0.137
No	10; 1087.56 (42.3)	10; 692.100 (26.9)	10; 2669.50 (30.8)	
^**1**^**SBP mmHg**,** n; n**^**pond**^(%)***				
Above recommended	19; 5865.415 (32.0)	25; 7195.728 (39.3)	21; 5260.787 (28.7)	0.119
Normal	12; 5563.274 (32.5)	11; 3688.602 (21.6)	21; 7860.682 (45.9)	
^**2**^**DBP mmHg**,** n; n**^**pond**^(%)***				
Above recommended	11; 4716.561 (30.5)	14; 4716.467 (30.5)	19; 6046.817 (39.1)	0.210
Normal	20; 6712.128 (33.6)	22; 6167.863 (30.9)	23; 7074.653 (35.5)	
**Fasting Blood Glucose mg/dL**,** n; n**^**pond**^(%)***				
Above recommended	8; 3507.176 (33.1)	14; 3507.091 (33.1)	17; 3567.595 (33.7)	0.109
Normal	23; 7921.513 (31.7)	22; 7377.239 (29.5)	26; 9674.812 (38.7)	
**Total cholesterol mg/dL**,** n; n**^**pond**^(%)***				
Above recommended	21; 7316.718 (30.9)	25; 8405.026 (35.5)	24; 7921.331 (33.5)	0.162
Normal	10; 4111.971 (34.5)	11; 2479.304 (20.8)	19; 5321.076 (44.7)	
^**3**^**LDL-c mg/dL**,** n; n**^**pond**^(%)***				
Above recommended	13; 4474.630 (26.2)	18; 6409.674 (37.6)	17; 6167.780 (36.2)	0.155
Normal	16; 5744.634 (33.8)	16; 4293.251 (25.3)	25; 6953.689 (40.9)	
^**4**^**HDL-c mg/dL**,** n; n**^**pond**^(%)***				
Below recommended	9; 626.18 (23.5)	10; 939.26 (35.2)	12; 1104.04 (41.4)	0.178
Normal	20; 2158.66 (32.5)	25; 2010.38 (30.3)	30; 2471.76 (37.2)	
**Triglycerides mg/dL**,** n; n**^**pond**^(%)***				
Above recommended	13; 4414.213 (27.5)	19; 5986.274 (37.4)	22; 5623.526 (35.1)	0.124
Normal	18; 7014.475 (35.9)	17; 4898.056 (25.1)	21; 7618.881 (39.0)	
^**5**^**CHDI**,** n; n**^**pond**^(%)**				
Low	71; 8651.06 (60.6)	36; 3262.72 (22.8)	26; 2372.88 (16.6)	**0.024**
Medium	40; 3888.88 (29.7)	48; 4234.94 (32.3)	44; 4976.42 (38.0)	
High	21;1927.96(14.8)	49; 4679.84 (35.8)	63; 6459.46 (49.4)	

^1^SBP: systolic blood pressure, ^2^DBP: diastolic blood pressure, ^3^LDL-c: Low-density lipoprotein, ^4^HDL-c: High-density lipoprotein, ^5^CHDI: Cardiovascular Health Diet Index – Low(1st tercile), Medium(2nd tercile) e High(3nd tercile). * Pearson’s Chi-square tests; **n = 398; *** *n* = 111

**Fig. 2:**
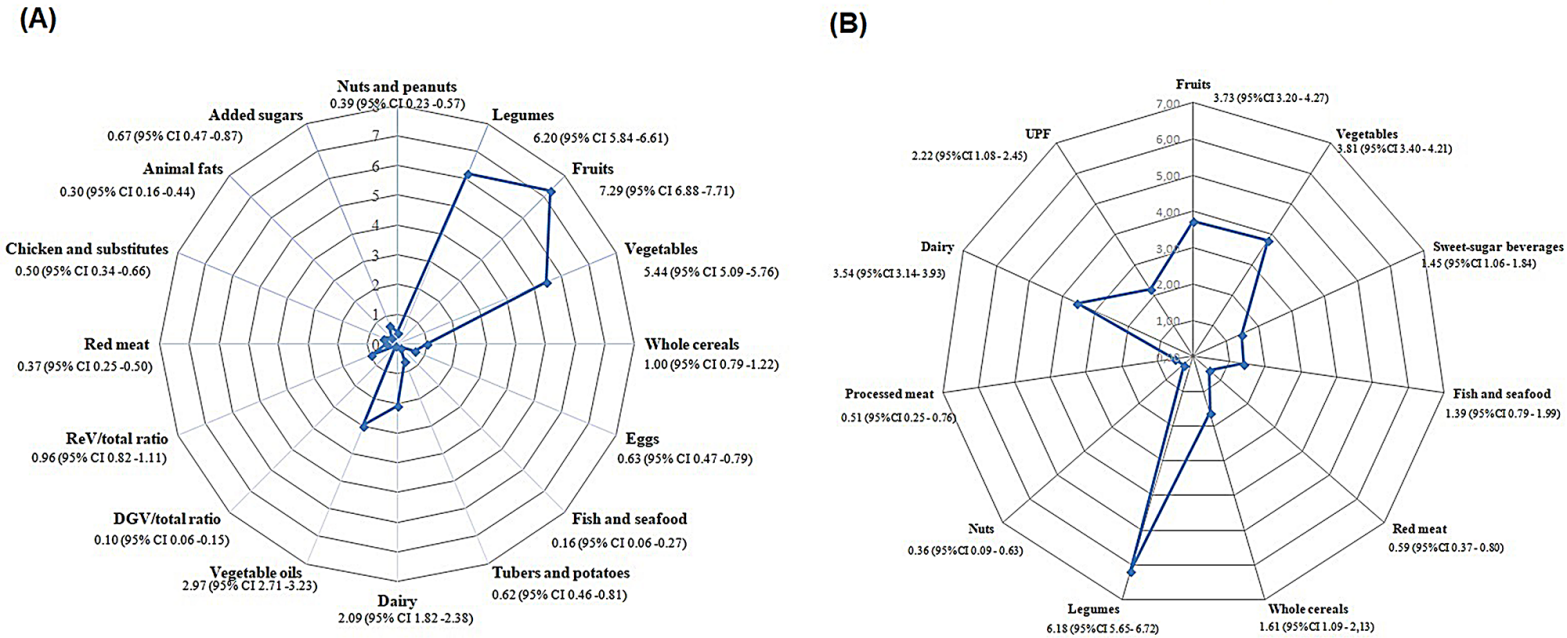
**a)** Means and standard deviations of the scores for components of the Planetary Health Diet Index (PHDI). Source: Oliveira Neta et al., 2023 [43]. **b)** Averages and standard deviations of the scores pertaining to components of the Cardiovascular Health Diet Index (CHDI) by food groups with their respective confidence intervals, according to the dietary intake of participants in the Brazuca-Natal Study, Brazil (2019–2020). Source: Authors’ elaboration (2024).*DGV/total: the ratio between the energy intake from dark green vegetables (numerator) and the total vegetable intake (denominator) multiplied by 10. **ReV/total: the ratio between the energy intake from red and orange vegetables (numerator) and the total vegetable intake (denominator) multiplied by 10. ***UPF: ultra-processed foods

The mean PHDI was 29.4 points (95% CI 28.04:30.81), on a total score ranging from 0 to 150, with higher scores for fruits, vegetables, legumes, oils, and dairy, respectively (Fig. 2A). The mean CHDI was 32.63 points (95% CI 31.50:33.78), on a total score ranging from 0 to 110, with higher scores for legumes, fruits, vegetables, and dairy, respectively (Fig. 2B).

.

The final score of the CHDI was positively associated with the PHDI (β = 0.12, 95% CI 0.31–0.54), as well as with fruits (β = 0.60, 95% CI 0.16–1.04), vegetables (β = 1.22, 95% CI 0.65–1.75), and legumes (β = 0.64, 95% CI 0.08–1.21). Conversely, the PHDI was negatively associated with the consumption of UPF (β = -1.28, 95% CI -2.24:-0.33). No significant associations were found for fish and seafood, red meats, sugar-sweetened beverages, whole cereals, nuts, processed meats, and dairy products (Table 2).

**Table 2:** Association between the Planetary Health Diet Index (PHDI), Cardiovascular Health Diet Index (CHDI), and their components consumed by adults and elderly individuals from the Brazuca-Natal study, Brazil (2019–2020)

Variables	β	CI 95%	*p*-value*	*R*²
^1^CHDI	0.12	0.31:0.54	**0.050**	0.482
Fruits	0.60	0.16:1.04	**0.010**	
Vegetables	1.22	0.65:1.75	**0.001**	
Fish and seafood	0.061	-0.53:0.65	0.832	
Red meat	-0.35	-1.13:0.43	0.366	
^2^SSB	0.11	-0.39:0.60	0.666	
Whole cereals	0.49	-0.09:1.06	0.092	
Legumes	0.64	0.08:1.21	**0.028**	
Nuts	0.96	-0.12:2.03	0.080	
Processed meat	0.05	-0.80:0.90	0.901	
Dairy	-0.09	-0.54:0.37	0.704	
^2^UPF	-1.28	-2.24:-0.33	**0.010**	

*Linear regression adjusted for age, income, and BMI. ^1^CHDI: Cardiovascular Health Diet Index. ^2^SSB: Sugar-sweetened beverages. ^2^UPF: ultra-processed foods

Table 3 quantitatively provides, through the NOVA score, the UPF consumed and their percentage of caloric contribution by participants of the Brazuca-Natal Study, Brazil (2019–2020). It can be observed that the higher the quantitative participation of UPF in the diet, the more significant their caloric contribution.

**Table 3:** Contribution of ultra-processed foods to the diet, according to the NOVA score of ultra-processed food consumption. Brazuca Study – Natal, Brazil (2019–2020)

Number of ^1^UPF points present in the diet	*N* (%)	Percentage (95% CI) of the caloric contribution of UPF to the total energy consumed
0	5091.86 (12.6)	0
1	11683.12 (28.9)	9.78 (8.07–11.48)
2	8338.04 (20.6)	13.57 (11.27–15.88)
3	7036.18 (17.4)	19.01 (17.56–20.48)
4	4070.08 (10.1)	23.55 (20.62–26.49)
5	2026.82 (5.0)	31.93 (17.08–46.78)
6	1697.24 (4.2)	38.49 (30.52–46.46)
7	510.82 (1.3)	39.01 (24.56–53.45)

^1^UPF: Ultra-processed food

**Fig. 3:**
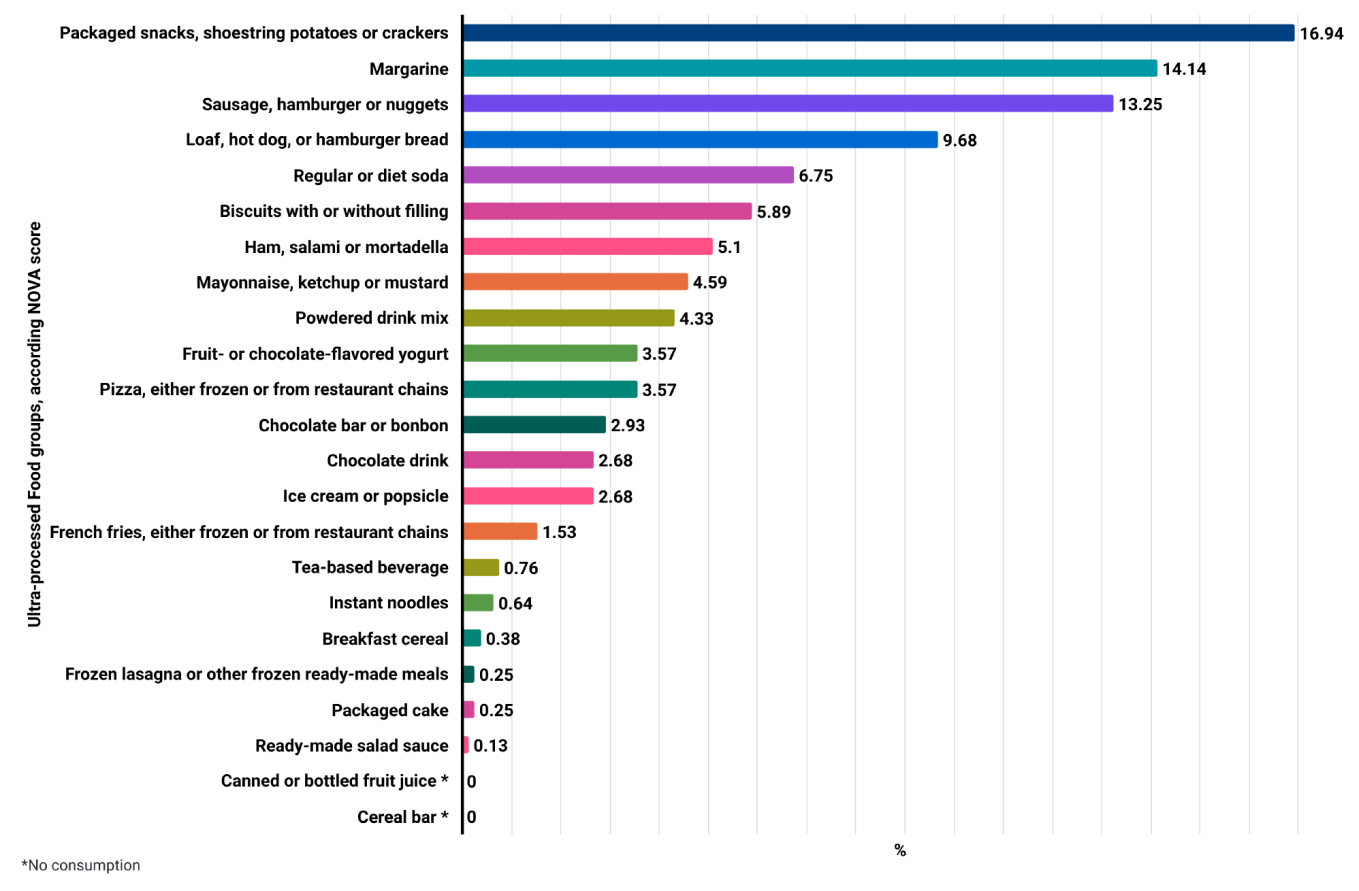
Frequency (%) of consumption of foods included in the NOVA score of UPF consumption according to participants’ dietary intake in the Brazuca-Natal Study, Brazil (2019–2020). Source: Authors’ elaboration (2024)

In Fig. 3, the frequency of ultra-processed food consumption according to the NOVA score shows a higher frequency of consumption of ultra-processed foods such as packaged snacks, shoestring potatoes, and crackers (16.94%), followed by margarine (14.14%) and processed meats (13.25%) among adults and elderly individuals in the Brazuca-Natal study.

From the results obtained in Table 4, it can be inferred that when using the linear regression model adjusted for age, income, and BMI, individuals had a negative association with SBP (mmHg) (β -0.14, 95% CI -0.25 to -0.01), total cholesterol (mg/dL) (β -0.80, 95% CI -1.31 to -0.46), and LDL-c (mg/dL) (β -0.10, 95% CI -0.19 to -0.08) with *p* < 0.05. No association was found for DBP, fasting blood glucose (mg/dL), HDL-c (mg/dL), and triglycerides (mg/dL).

**Table 4:** The association between the Planetary Health Diet Index (PHDI) and cardiometabolic risk factors among adults and elderly individuals in the Brazuca-Natal Study, Brazil (2019–2020) is depicted in the analysis

Variables	β	95% CI	*p*-value*	*R* ^2^
Model 1				
^1^DBP (mmHg)	0.46	0.02:0.90	**0.041**	0.427
^1^SBP (mmHg)	-0.14	-0.25:-0.01	**0.016**	
Fasting Blood Glucose (mg/dL)	0.12	-0.26:0.49	0.514	
Total cholesterol (mg/dL)	-0.80	-1.31:-0.46	**0.021**	
^3^LDL-c (mg/dL)	-0.10	-0.19:-0.08	**0.026**	
^4^HDL-c (mg/dL)	-0.02	-0.45:0.49	0.928	
Triglycerides (mg/dL)	-0.21	-0.55:0.14	0.227	
**Model 2**				
Diabetes mellitus	-5.58	-10.20:-0.95	**0.013**	
Dyslipidemia	-4.19	-8.48:-0.09	**0.044**	0.474
Arterial Hypertension	-1.63	-6.00:2.78	0.450	

**Linear regression adjusted for age, income, and BMI. ^1^SBP: systolic blood pressure, ^2^DBP: diastolic blood pressure, ^3^LDL-c: Low-density lipoprotein, ^4^HDL-c: High-density lipoprotein

We also observed in Table 4 that the sustainability index of the diet is 5.58 points lower for individuals with type 2 diabetes (95% CI -10.20:-0.95), and similarly, people with dyslipidemia have 4.19 points less (95% CI -8.48:-0.09) (*p* < 0.05). No association was found between PHDI and self-reported hypertension (R²=0.47).

## Discussion

Planetary health is a global concern focused on the dynamic and systemic relationships between human action in ecosystems and how it affects human health and well-being [52]. In this study, we assessed the association between adherence to the EAT-Lancet diet (PHDI) with a dietary index of cardiovascular health, the CHDI, and cardiometabolic risk factors. We found that PHDI was significantly associated with lower SBP, total cholesterol, and LDL-c levels, as well as with individuals who self-reported having type 2 diabetes and dyslipidemia, who had lower scores in adherence to the EAT-Lancet diet. Conversely, individuals with higher scores on the PHDI were associated with better cardiovascular health assessed by the CHDI and its components (fruits, vegetables, and legumes), demonstrating that a sustainable diet can benefit the planet and human health.

Although the concepts of healthy and sustainable diets have long been addressed separately [11, 31], in 2021, the definition of a healthy diet expanded to encompass not only the promotion of human health and disease prevention but also the preservation of planetary health. In this context, the latest literature on healthy and sustainable diets considers them interconnected and inseparable concepts [53]. Therefore, this discussion is organized in this way: Firstly, we address the harmony between sustainability indicators and the health of diets and the mechanisms that could explain this interaction. We also explore the food groups that exert the most notable influence on this synergy, emphasizing the difficulties encountered by countries in the Global South when embracing a diet that is both healthy and sustainable. Finally, we emphasize how this work helps us understand that there is a vital synergy between dietary practices that respect the ecological limits of our planet and the well-being of the human population, according to the emerging principles of planetary health.

Evidence aimed at developing indices to assess the sustainability of diets emerges as a strategy to identify dietary patterns that promote human health and exert low pressure on planetary boundaries [24, 54–57]. These studies include research to estimate the environmental impact of food consumption in various world regions [14, 58–61]. Evidence suggests that sustainable diets can bring benefits in preventing CVDs and cardiometabolic risk profiles [27, 60–62]. Knuppel et al. found that high adherence to the EAT-Lancet score was associated with lower risks of ischemic heart disease (28%] and diabetes (59%) [62). Seconda et al. (2020) and Stubbendorff et al. (2022) obtained a similar relationship between this adherence and the risk of cardiovascular mortality [58, 63]. In Brazil, Cacau et al. (2023) found lower blood pressure levels, total cholesterol, and low-density lipoprotein cholesterol in individuals with higher adherence to sustainable diets [26]. Indeed, our results showed this positive synergy between adhering to a sustainable diet concerning the benefits in individuals with T2DM and dyslipidemia and cardiometabolic parameters.

The mechanisms driving the synergy between having a sustainable diet and cardiovascular health can largely be attributed to three processes: Firstly, a sustainable diet is rich in fiber, mainly derived from fruits, vegetables, and legumes, which play a prominent role in the dietary treatment of CVDs and T2DM [59, 64]. Evidence indicates that soluble fibers influence the production of glucagon-like peptide-1 (GLP-1), can delay gastric emptying, reduce glucose entry into the bloodstream, and thus modulate its absorption [65, 66].

The second mechanism is maintaining a healthy weight provided by a sustainable diet, which contributes to preventing chronic diseases in adulthood, as obesity is considered an intermediate risk marker for these outcomes [2, 29, 64, 67]. In this regard, the diet proposed by the EAT-Lancet Commission consists primarily of plant-based foods, low amounts of animal-derived foods, and fats. It discourages the intake of refined grains and added sugars [11], fostering not only short-term weight loss but also the sustained maintenance of a healthy weight, thereby reducing the risks of CVDs and T2DM in the long term [64].

Lastly, improving intestinal microbiota composition induced a the sustainable diet can positively contribute to metabolic health [68]. The replacement of animal-derived proteins with plant sources has been associated with anti-inflammatory effects and increased presence of health-promoting bacteria in the body, such as *Bifidobacterium* and *Lactobacillus*, while reducing those considered pathogenic, such as *B. fragilis* and *Clostridium perfringens*, thereby increasing susceptibility to various diseases [68, 69].

The protective effect of fruits and vegetables aligns positively with a healthy and sustainable diet [4, 11]. The WHO recommends consuming 400 g of fruits and vegetables daily to promote health and reduce chronic diseases [4, 13, 16, 55, 64]. These health benefits are associated with their high fiber content and potential prebiotic and phytonutrient properties [70]. Regarding sustainability, fruits, and vegetables are foods that align with lower environmental impacts; however, priority should be given to consuming seasonal fruits and vegetables, locally produced with minimal pesticide use and involved in a short transportation chain to enhance their sustainable characteristics [11, 71].

Legumes are an attractive strategy to encourage the adoption of this diet due to their high fiber and protein content and, in terms of sustainability, their low cost and reduced environmental impact [72, 73]. However, changes in dietary patterns have been observed in populations, especially in Brazilians, including a reduction in the consumption of beans, traditionally the most consumed legumes in the country [35]. This is partly due to the time-consuming nature of their preparation, making them easily substituted for ultra-processed products [72]. Current evidence shows that this behavior may be harmful in terms of increasing the risk of developing CVD, including outcomes such as hypertension, dyslipidemia, diabetes, and obesity [73].

Among the foods that stand out negatively in the synergy between a good diet for health and for the planet is UPF, characterized by their degree of processing, high in ‘empty’ calories (fats and sugars), which become attractive to consumers for their convenience, low cost, and high palatability [46]. It is important to note that they also promote poor diet quality and the global spread of CVD [32, 44, 76, 77] due to their higher glycemic load and lower brain satiety signaling in the gut [74, 75]. Sustainability emerges as an additional aspect of the challenge of consuming UPF, as evidence shows that the diet’s growing negative environmental impact (CO2, land use, water, and energy) comes from these foods [33, 34, 76–78].

Our results showed that among the most consumed UPF by participants of the Brazuca-Natal study were calorically dense items belonging to the group of ‘packaged snacks, shoestring potatoes, crackers, and ‘Margarine’. According to the NOVA score, UPF are linked to excessive caloric intake [44], and this overconsumption of calories can indirectly be considered as promoting adverse effects on the environment [32]. Thus, simply restricting caloric intake from UPF may reduce intermediate risk factors leading to CVD and emerge as a direct strategy to mitigate the environmental impacts associated with diets [82].

Historically, meat is one of the most consumed foods by Brazilians [45] despite its reduction in consumption due to socioeconomic reasons recorded in the NDS-HBS (2017–2018), it is still a traditionally highly consumed food that threatens the harmony between a healthy and environmentally friendly diet [35, 79]. A relevant issue contributing to this imbalance is its nutritional composition, as it is rich in saturated fat and often undergoes some form of processing, increasing its sodium content and potentially leading to CVD [7, 30, 67]. Another aggravating factor is that Brazil is considered the largest producer and exporter of meat globally, associated with large areas of deforestation for cattle ranching and high greenhouse gas emissions from these animals, contributing to climate change and biodiversity loss [79, 80].

We emphasize the importance of considering the quality of this meat, as the consumption of processed meats by economically vulnerable families is frequent due to their lower market value [80]. In our study, the consumption of 13.25% of foods from the ‘Sausage, Hamburger, and Nuggets group and 5.10% from the ‘Ham, Salami, and Mortadella group is concerning, and this results from inequalities in accessibility, as food prices are one of the most significant barriers to dietary choices, compromising both human and planetary health [79–81]. As a solution, it is recommended that this consumption be moderated and replaced with dairy products, vegetables, eggs, and fish [11, 43, 56].

Regarding fish consumption, there is a conflict between dietary recommendations and environmental sustainability. While fish are recognized for their cardioprotective benefits due to their protein content, micronutrients, and omega-3 fatty acids, evidence indicates that marine stocks are declining, and there are not enough fish available to meet global dietary guidelines, primarily due to unsustainable fishing practices [82–84]. It’s important to highlight that opting for fish consumption remains beneficial in comparison to the intake of red meats and processed meats [4]. Another food item facing the duality of not having sustainable production yet being healthy due to being an excellent source of B vitamins is milk. Its production stimulates cattle farming, a significant producer of greenhouse gas emissions, leading the EAT-Lancet diet to suggest its moderate consumption [11, 71].

Underdeveloped countries like Brazil, Gambia, India, and Mexico have been gaining prominence internationally concerning the analysis of sustainable diets [24, 55, 85, 86]. However, there are some barriers to these dietary changes, such as measuring adherence in countries with continental dimensions and heterogeneous territories, as in the case of Brazil, which is marked by socioeconomic inequalities in food consumption across its various regions [26, 87, 88]. The discourse on a healthy and sustainable diet goes beyond determining which foods to consume in what quantities, as it involves food sovereignty and nutritional security in a food system that was not structured to promote public health and environmental preservation [71].

Another significant current challenge is the influence the media has historically exerted on culture, leading to increasing standardization of dietary patterns and reducing the diversity of available food options. Traditional and culturally significant food products have gradually given way to highly processed items [89–91]. As a result, more unhealthy foods are produced than healthy ones, making them less accessible while the unhealthy ones become the cultural norm [71], impacting the sustainability of any diet as it is influenced by sociocultural factors such as food preferences, attitudes, values, social structures, and cultural practices [92].

In our study population, we found that adhering to a sustainable diet was significantly associated with the index that globally evaluates cardiovascular health (CHDI) and cardiometabolic outcomes (lower SBP, total cholesterol, and LDL-c levels), and individuals with type 2 diabetes mellitus and dyslipidemia had a lower score in adherence to the EAT-Lancet diet. These results align the ecological boundaries of our planet with the well-being of the human population, according to the emerging principles of planetary health.

Although it is possible to identify the outcomes above in our study population, it is essential to realize that this may not represent a significant proportion of the total population of Brazil, as this is a cross-sectional analysis, which does not allow for the assessment of causality, such as its ability to predict death and/or disease. However, given that Brazuca-Natal is a follow-up study, it encourages the development of further research to continue this discussion. As limitations, we had an interruption in data collection due to the advent of the COVID-19 pandemic, which was one of the reasons for only using just one 24-hour Record, reflecting only the current diet of the research participants. This did not allow us to check the frequency of food consumption on other dates. This was overcome in the complex statistical analysis that allowed our sample to be representative for the entire city of Natal-RN.

As strengths of our study, we highlight the originality in assessing the association between adherence to the EAT-Lancet diet and a cardiovascular health diet index (CHDI) that considers the consumption of UPF and cardiometabolic risk factors. To some extent, this index addresses the deficiency in existing indices that evaluate adherence to sustainable diets, which do not account for UPF consumption in their structure, thus providing the groundwork for future discussions on the topic. Additionally, the 24-hour dietary recall (R24h) for data collection and analysis was a distinguishing factor, as it exhibits high validity at the individual level compared to methods that rely on food frequency, which may introduce biases such as the finite list of foods and self-reporting [93].

## Conclusions

In conclusion, the results of our analysis suggest that adopting sustainable diets, especially the EAT-Lancet diet, positively correlates with improvements in key health indicators. These findings reinforce the hypothesis that there is a vital synergy between dietary practices that respect the ecological limits of our planet and the well-being of the human population, aligning with the emerging principles of planetary health. Furthermore, our findings indicated that higher consumption of UPF is associated with lower adherence to sustainable diets.

Our findings support the idea that a diet can benefit for both human cardiovascular health and the planet simultaneously as long as the population has access to various food production, processing, and marketing modes. In terms of consumption, the inseparable integration between promoting healthy and sustainable dietary habits is considered vital, ensuring that principles are harmonized and incorporated into nutritional recommendations. In this regard, the State’s intervention in food policies is highlighted as an essential factor in solidifying the practice of sustainable eating.

## Data Availability

No datasets were generated or analysed during the current study.
